# Risk factors for community-acquired pneumonia among inpatients with mental disorders in a tertiary general hospital

**DOI:** 10.3389/fpsyt.2022.941198

**Published:** 2022-07-22

**Authors:** Jingjing Han, Meiyu Shen, Qirong Wan, Zhihua Lv, Ling Xiao, Gaohua Wang

**Affiliations:** ^1^Department of Infection Control, Renmin Hospital of Wuhan University, Wuhan, China; ^2^Department of Mental Health Center, Renmin Hospital of Wuhan University, Wuhan, China; ^3^Department of Clinical Psychology, Renmin Hospital of Wuhan University, Wuhan, China; ^4^Department of Clinical Laboratory, Renmin Hospital of Wuhan University, Wuhan, China; ^5^Insititute of Neuropsychiatry, Renmin Hospital of Wuhan University, Wuhan, China; ^6^Department of Psychiatry, Renmin Hospital of Wuhan University, Wuhan, China

**Keywords:** mental disorders, community-acquired pneumonia, general hospitals, inpatients, risk factors

## Abstract

**Introduction:**

Community-acquired pneumonia (CAP) is an important cause of hospitalization and death in patients with mental disorders. It is critical to understand the risk factors of CAP and determine prevention strategies to reduce CAP. The aim of this study is to explore the characteristics of inpatients with mental disorders who have CAP and analyze the risk factors.

**Methods:**

This retrospective study included 16,934 inpatients with mental disorders who were admitted for the first time to a tertiary general hospital between January 2017 and July 2021 (excluding January 2020–May 2020). Risk factors for CAP were identified by logistic regression analysis after propensity score matching (PSM, 1:4) for age, gender, and BMI.

**Results:**

The CAP rate of inpatients with mental disorders was 1.78%. Inpatients who had CAP had a significantly prolonged hospital stay, and were more often admitted to a closed ward or the ICU. After PSM, the multivariable analysis revealed that clozapine use (OR = 3.212, 95% CI = 1.744–5.915, *P* < 0.001), schizophrenia spectrum disorder (OR = 2.785, 95% CI = 1.684–4.607, *P* < 0.001), alcohol consumption (OR = 2.549, 95% CI = 1.586–4.096, *P* < 0.001), cardiovascular disease (OR = 2.299, 95% CI = 1.362–3.879, *P* = 0.002), Charlson comorbidity index (CCI) ≥ 3 (OR = 2.092, 95% CI = 1.342–3.260, *P* = 0.001), organic mental disorder (OR = 1.941, 95% CI = 1.194–3.156, *P* = 0.007), antipsychotic drug use (OR = 1.886, 95% CI = 1.312–2.711, *P* = 0.001), unmarried status (OR = 1.720, 95% CI = 1.164–2.541, *P* = 0.006) and junior high school education (OR = 1.591, 95%CI = 1.010–2.508, *P* = 0.045) were independent risk factors for CAP in inpatients with mental disorders.

**Conclusion:**

CAP was common in inpatients with mental disorders. Patients with mental disorders have unique risk factors for CAP. Further research is required to explore the relationship and mechanism between different mental disorders, antipsychotic drugs and CAP.

## Introduction

Mental disorders are a group of diseases that manifest as cognitive, emotional, and behavioral disturbances. The global burden of mental disorders has increased in recent years (1). Mental disorders account for 14.3% of all deaths worldwide, and around 8 million people with mental disorders die each year (2). Most of the reductions in life expectancy in people with mental disorders appear to be due to somatic comorbidities or other natural causes of death such as cardiovascular disease (CVD) and pneumonia (3).

Community-acquired pneumonia (CAP), which develops in people outside of healthcare settings, is a serious disease with a potentially poor long-term prognosis. CAP is particularly prevalent in developing countries and is associated with high morbidity, hospitalization, and mortality rates (4). Epidemiological studies have found that patients with mental disorders have a significantly increased risk of developing CAP (5). CAP is an important cause of hospitalization and death in patients with mental disorders (6). Patients with mental disorders such as schizophrenia and depression have a generally poorer prognosis after developing CAP, such as higher occupancy rates in the intensive care unit (ICU) (7).

Risk factors for CAP in the general population include advanced age, male gender, smoking, alcoholism, respiratory disease, dementia, and various comorbidities (8). People with mental disorders are more likely to use antipsychotic drugs, have unhealthy lifestyles, show poor adherence to treatment, and have poor access to medical services (3). All of these factors may affect the occurrence of CAP. To date, few published studies have evaluated the epidemiology of CAP and the risk factors for CAP in inpatients with mental disorders. A retrospective analysis of 2,209 patients with schizophrenia hospitalized in Tokyo, Japan, found that 101 patients (4.6%) were diagnosed with pneumonia on admission (9). Furthermore, advanced age (≥50 years-old), body mass index (BMI) <18.5 kg/m^2^, smoking, use of atypical antipsychotic drugs, and high-dose antipsychotic drugs were risk factors for pneumonia on admission in patients with schizophrenia (9). The above study mainly focused on patients in specialized psychiatric hospitals and on schizophrenia. There are few reports describing the epidemiological characteristics of CAP among inpatients with mental disorders in general hospitals.

Our hospital is a tertiary general hospital with the largest number of psychiatric beds in China (350 beds, including 225 beds on open wards and 125 beds on closed wards). Our hospital also has high-level respiratory, infectious disease, intensive care medicine, and radiology departments, which allows patients with suspected CAP to be diagnosed and treated in a timely manner. The aim of this cross-sectional study was to investigate the epidemiological characteristics of CAP among inpatients with mental disorders admitted to a general hospital in China.

## Methods

### Study design and patients

This retrospective study included inpatients diagnosed with mental disorders at Renmin Hospital of Wuhan University between January 2017 and July 2021. The Ethics Committee of Renmin Hospital of Wuhan University approved this study (WDRY2021-KS069) and waived the requirement for informed consent due to the retrospective nature of the analysis.

The inclusion criteria were as follows: (1) admitted to the hospital with a primary diagnosis of a mental disorder or a mental disorder with pneumonia/pulmonary infection; (2) hospitalized for >48 h; and (3) aged ≥ 18 years-old. The diagnoses of the mental disorders were made in accordance with the tenth revision of the International Classification of Mental Disorders (ICD-10, codes F00–F99) (WHO, 1992). Patients were excluded from the final analysis if any of the following criteria were met: (1) hospital-acquired pneumonia (HAP); (2) died or discharged from the hospital within 48 h of admission; (3) admitted during the COVID-19 pandemic (January 2020–May 2020); (4) readmitted after a first admission during the study period; and (5) incomplete data.

### Data collection and definition

The following baseline data were extracted from the medical records: age, gender, education level, marital status, smoking history, history of alcohol consumption, history of mental disorder (including type, age at onset, disease course, and current treatment strategy), BMI, comorbidities, type of ward admitted to (closed or open), the requirement for ICU admission, the requirement for mechanical ventilation, duration of hospitalization and survival.

The inpatients with mental disorders were divided into a CAP group and a non-CAP group according to whether comorbid CAP was present. The diagnosis of CAP was made if the patient met all three of the following criteria (10): (1) onset in the community; (2) any one of the following pneumonia-related clinical manifestations: (i) newly developed cough or expectoration or exacerbation of existing respiratory disease symptoms with or without purulent sputum, chest pain, dyspnea, or hemoptysis; (ii) fever; (iii) signs of pulmonary consolidation and/or moist rales; (iv) peripheral blood leukocytes > 10 × 10^9^/L or <4 × 10^9^/L with or without a left shift; (3) chest imaging demonstrated new patchy infiltrates, lobar or segmental consolidation, ground-glass opacities, or interstitial changes with or without pleural effusion. They were excluded if other lung diseases (such as pulmonary tuberculosis, lung tumor, non-infectious pulmonary interstitial disease, pulmonary edema, atelectasis, pulmonary embolism, pulmonary eosinophilic infiltration, or pulmonary vasculitis) were present.

### Statistical analysis

The data were analyzed using SPSS 22.0 (IBM Corp., Armonk, NY, USA). Measurement data were tested for normality using the Kolmogorov-Smirnov method (sample size ≥ 50) or the Shapiro-Wilk method (sample size < 50). Normally-distributed measurement data are presented as the mean ± standard deviation (SD) and were compared between groups using the *t*-test for independent samples. Non-normally-distributed measurement data are shown as median [interquartile range (IQR)] and were compared between groups using the Mann–Whitney U test. Non-normally-distributed data were compared between multiple groups using the Kruskal–Wallis test. Count data are presented as frequency (percentage) and were analyzed using the chi-squared test or Fisher's exact test. Propensity score matching (PSM) was carried out using R 4.1.1 software (R Foundation for Statistical Computing, Vienna, Austria), and 1:4 matching was performed using variables such as age, gender, and BMI. Factors associated with CAP in patients with mental disorders were explored using univariate and multivariable logistic regression analyses. The presence or absence of CAP was used as the outcome variable, and the demographic/laboratory indicators were used as the independent variables. Variables with a *p* < 0.05 in the univariate analysis were entered into the multivariate analysis (enter method). Odds ratios (ORs) and 95% confidence intervals (95% CIs) were calculated. Multicollinearity was considered if Tolerance was <0.2 or the variance inflation factor (VIF) was >10. No multicollinearity was observed in the multivariable model. *P* < 0.05 was considered statistically significant.

## Results

### Baseline characteristics of the study participants

A total of 25,891 patients with mental disorders were admitted during the study period. After the exclusion of patients under the age of 18 years-old (*n* = 3,853), patients with a hospital stay of <48 h (*n* = 449), patients with HAP (*n* = 194), repeat admissions (*n* = 4,419) and patients with incomplete pre-admission clinical data (*n* = 42), the final analysis included 16,934 patients. The baseline clinical characteristics of the study participants before PSM are shown in Supplementary Table 1. The 301 patients (168 men, 55.81%) in the CAP group had a median age of 50.00 (IQR, 33.00–62.00) years, and the 16,633 patients (7,063 men, 42.46%) in the non-CAP group had a median age of 31.00 (IQR, 24.00–48.00) years.

### Incidence of CAP

The incidence rate of CAP was 1.78% overall, 8.44% in patients with organic mental disorders, 2.80% in patients with schizophrenia spectrum disorders, 1.04% in patients with mood-affective disorders, and 1.28% in patients with other mental disorders. The incidence rate of CAP increased with age (1.06% for those aged 18–44 years-old, 2.68% for those aged 45–64 years old, and 6.86% for those aged ≥ 65 years-old), was higher in men than in women (2.32 vs. 1.37%) and higher in patients with a BMI ≤ 18.5 kg/m^2^ than in those with a BMI > 18.5 kg/m^2^ (3.90 vs. 1.65%).

### Baseline characteristics of the study participants after PSM

The propensity score-matched data consisted of 1,495 patients and included 299 patients with CAP and 1,196 patients without CAP. There were significant differences in education, smoking, alcohol consumption, type of mental disorder, age at onset of the mental disorder, duration of the mental disorder, duration of treatment for the mental disorder, duration of treatment with antipsychotic drugs, use of antipsychotic drugs, use of clozapine, use of cholinesterase inhibitors, CCI and incidences of comorbid diseases (cerebrovascular disease, CVD, diabetes mellitus) between the CAP group and non-CAP group after PSM (Table 1).

**Table 1:** Baseline characteristics of the study participants after propensity score matching.

**Characteristic**	**CAP group (*****n*** = **299)**	**Non-CAP group (*****n*** = **1,196)**	***P***
Age (years), median (IQR)	50.00 (33.00, 62.00)	50.00 (33.00, 62.00)	0.967
Gender, Male, *n* (%)	166 (55.52%)	665 (55.60%)	0.979
Education, *n* (%)			0.014
Undergraduate and above	41(13.71%)	250(20.90%)	
College	9 (3.01%)	62 (5.18%)	
High school or secondary school	93 (31.10%)	361 (30.18%)	
Junior high school	87 (29.10%)	291 (24.33%)	
Primary school/illiterate	69 (23.08%)	232 (19.40%)	
Marital status, *n* (%)			0.068
Unmarried	83 (27.76%)	267 (22.32%)	
Married	181 (60.54%)	808 (67.56%)	
Widowed or divorced	35 (11.71%)	121 (10.12%)	
Body mass index, ≤18.5 kg/m^2^, *n* (%)	35 (11.71%)	134 (11.20%)	0.806
Smoking, *n* (%)	75 (25.08%)	229 (19.15%)	0.023
Alcohol consumption, *n* (%)	53 (17.73%)	114 (9.53%)	<0.001
Type of mental disorders, *n* (%)			<0.001
Organic mental disorder	59 (19.73%)	131 (10.95%)	
Schizophrenia spectrum disorder	100 (33.44%)	192 (16.05%)	
Mood affective disorder	97 (32.44%)	586 (49.00%)	
Other	43 (14.38%)	287 (24.00%)	
Age at mental disorder onset (years), median (IQR)	36.00 (24.00, 57.00)	41.00 (27.00, 58.00)	0.028
Duration of mental disorder (years), median (IQR)	4.00 (0.30, 10.00)	2.00 (0.20, 8.00)	0.002
Family history of mental disorder, *n* (%)	36 (12.04%)	155 (12.96%)	0.670
Duration of treatment (years), median (IQR)	1.00 (0.00, 7.00)	0.50 (0.00, 3.00)	<0.001
Duration of antipsychotic drug use (years), median (IQR)	0.20 (0.00, 5.00)	0.00 (0.00, 1.00)	<0.001
Poor adherence to recent therapy^a^, *n* (%)	37 (12.37%)	147 (12.29%)	0.969
Number of antipsychotic drugs currently used, *n* (%)			<0.001
0	159 (53.18%)	881 (73.66%)	
1	79 (26.42%)	213 (17.81%)	
2/3/4	61 (20.40%)	102 (8.53%)	
Current use of clozapine, *n* (%)	64 (21.40%)	56 (4.68%)	<0.001
Cholinesterase inhibitor use, *n* (%)	23 (7.69%)	50 (4.18%)	0.012
Charlson comorbidity index, *n* (%)			<0.001
0–1 points	145 (48.49%)	758 (63.38%)	
2 points	38 (12.71%)	172 (14.38%)	
3 points and above	116 (38.80%)	266 (22.24%)	
Cerebrovascular disease, *n* (%)	86 (28.76%)	195 (16.30%)	<0.001
Cardiovascular disease, *n* (%)	41 (13.71%)	47 (3.93%)	<0.001
Diabetes mellitus, *n* (%)	39 (13.04%)	65 (5.43%)	<0.001

a *Failure to take the medication regularly or medication discontinued without authorization during the past 1 month*.

*CAP, community-acquired pneumonia; IQR, interquartile range*.

### Outcomes of inpatients with mental disorders who had comorbid CAP

The patient outcomes were analyzed after PSM. Inpatients with mental disorders who had comorbid CAP had a significantly prolonged hospital stay, were more often admitted to a closed ward or the ICU, and were more often treated with mechanical ventilation than those without CAP (*P* < 0.001 for all parameters). However, there was no significant difference in mortality rate between the two groups (Table 2).

**Table 2:** Ward of admission and outcomes of inpatients with mental disorders stratified according to the presence/absence of comorbid community-acquired pneumonia.

**Parameter**	**CAP group (*****n*** = **299)**	**Non-CAP group** **(*****n*** = **1,196)**	***P***
Ward admitted to, *n* (%)			<0.001
Open ward	149 (49.83%)	952 (79.60%)	
Closed ward	110 (36.79%)	239 (19.98%)	
Intensive care unit	40 (13.38%)	5 (0.42%)	
Outcome, n (%)			0.114^a^
Clinical improvement	295 (98.66%)	1,191 (99.58%)	
No clinical change	2 (0.67%)	2 (0.17%)	
Death	2 (0.67%)	3 (0.25%)	
Mechanical ventilation, n (%)			<0.001^a^
No	294 (98.33%)	1,196 (100.00%)	
Yes	5 (1.67%)	0 (0.00%)	
Hospital stay (days), median (IQR)	15.00 (10.00, 24.00)	13.00 (9.00, 19.00)	<0.001

a *Fisher's exact probability method*.

*CAP, community-acquired pneumonia; IQR, interquartile range*.

Among the patients with CAP, hospital stay was significantly longer in those with schizophrenia or mood-affective disorder than in those with organic mental disorders or other types of mental illness (*P* = 0.039) (Table 3). Prognosis and length of hospital stay were comparable between patients admitted to open wards, closed wards, and the ICU. However, only patients admitted to the ICU received mechanical ventilation (Table 4).

**Table 3:** Ward of admission and outcomes of inpatients with mental disorders and community-acquired pneumonia stratified according to the type of mental disorder.

**Parameter**	**Organic mental disorder (*****n*** = **59)**	**Schizophrenia spectrum disorder** **(*****n*** = **100)**	**Mood affective disorder (*****n*** = **97)**	**Other** **(*****n*** = **43)**	***P***
Ward admitted to, *n* (%)					0.141
Open ward	31 (52.54%)	42 (42.00%)	55 (56.70%)	21 (48.84%)	
Closed ward	23 (38.98%)	38 (38.00%)	30 (30.93%)	19 (44.19%)	
Intensive care unit	5 (8.47%)	20 (20.00%)	12 (12.37%)	3 (6.98%)	
Outcome, *n* (%)					0.663^a^
Clinical improvement	57 (96.61%)	98 (98.00%)	97 (100.00%)	43 (100.00%)	
No clinical change	1 (1.69%)	1 (1.00%)	0 (0.00%)	0 (0.00%)	
Death	1 (1.69%)	1 (1.00%)	0 (0.00%)	0 (0.00%)	
Mechanical ventilation, *n* (%)					0.576^a^
No	59 (100.00%)	97 (97.00%)	95 (97.94%)	43 (100.00%)	
Yes	0 (0.00%)	3 (3.00%)	2 (2.06%)	0 (0.00%)	
Hospital stay (days), median (IQR)	14.00 (10.00, 18.00)	16.50 (11.00, 29.75)	17.00 (10.50, 28.00)	14.00 (10.00,18.00)	0.039

a *Fisher's exact probability method*.

*IQR, interquartile range*.

**Table 4:** Outcomes of inpatients with mental disorders and community-acquired pneumonia stratified according to ward of admission.

**Parameter**	**Admission to open ward (*****n*** = **149)**	**Admission to closed ward** **(*****n*** = **110)**	**Admission to intensive care unit (*****n*** = **40)**	***P***
Outcome, *n* (%)				0.218^a^
Clinical improvement	147 (98.66%)	109 (99.09%)	39 (97.50%)	
No clinical change	0 (0.00%)	1 (0.91%)	1 (2.50%)	
Death	2 (1.34%)	0 (0.00%)	0 (0.00%)	
Mechanical ventilation, *n* (%)				<0.001^a^
No	149 (100.00%)	110 (100.00%)	35 (87.50%)	
Yes	0 (0.00%)	0 (0.00%)	5 (12.50%)	
Hospital stay (days), median (IQR)	16.00 (11.00, 23.50)	16.00 (10.75, 26.50)	13.00 (9.00, 25.75)	0.788

a *Fisher's exact probability method*.

*IQR, interquartile range*.

### Logistic regression analyses of factors associated with CAP in inpatients with mental disorders

The multivariable analysis revealed that clozapine use, schizophrenia spectrum disorder, alcohol consumption, CVD, CCI ≥ 3 points, organic mental disorder, use of an antipsychotic drug, unmarried status, and junior high school education were independently associated with CAP in hospitalized patients with mental disorders (Table 5).

**Table 5:** Logistic regression analyses of factors associated with community-acquired pneumonia in inpatients with mental disorders.

**Variable**	**Multivariate analysis**	
	**OR (95% CI)**	***P***
Education		
Undergraduate and above	Reference	
College	0.829 (0.364, 1.888)	0.656
High school or secondary school	1.541 (0.992, 2.392)	0.054
Junior high school	1.591 (1.010, 2.508)	0.045
Primary school/illiterate	1.505 (0.927, 2.443)	0.099
Marital status		
Married	Reference	
Unmarried	1.720 (1.164, 2.541)	0.006
Widowed or divorced	1.109 (0.706, 1.743)	0.652
Smoking	0.984 (0.660, 1.468)	0.939
Alcohol consumption	2.549 (1.586, 4.096)	<0.001
Type of mental disorders		
Organic mental disorder	1.941 (1.194, 3.156)	0.007
Schizophrenia spectrum disorder	2.785 (1.684, 4.607)	<0.001
Mood-affective disorder	1.384 (0.906, 2.113)	0.133
Other	Reference	
Duration of mental disorder (years)	1.002 (0.982, 1.023)	0.837
Duration of treatment (years)	1.003 (0.958, 1.050)	0.894
Duration of antipsychotic drug use (years)	0.990 (0.944, 1.038)	0.684
Number of antipsychotic drugs currently used		
0	Reference	
1	1.886 (1.312, 2.711)	0.001
2/3/4	1.641 (0.913, 2.948)	0.098
Current use of clozapine	3.212 (1.744, 5.915)	<0.001
Cholinesterase inhibitor use	0.773 (0.416, 1.435)	0.414
Charlson comorbidity index		
0–1 points	Reference	
2 points	1.471 (0.928, 2.332)	0.101
3 points and above	2.092 (1.342, 3.260)	0.001
Cerebrovascular disease	1.336 (0.883, 2.021)	0.170
Cardiovascular disease	2.299 (1.362, 3.879)	0.002
Diabetes mellitus	1.533 (0.939, 2.503)	0.087

*OR, odds ratio; 95% CI, 95% confidence interval. All the variables listed in the table were the ones that were significant in the univariable analyses and entered the model. Otherwise, age, gender, body mass index, age at mental disorder onset, family history of mental disorder, and poor recent adherence to therapy did not enter the model*.

## Discussion

As far as we know, this is the first study to investigate the occurrence, risk factor analysis, and prognosis of CAP in hospitalized patients with mental disorders in a large general hospital.

The overall incidence of CAP in the patients with mental disorders admitted to this tertiary general hospital was 1.78%, which was higher than that reported for inpatients without mental disorders (11) but lower than that in a psychiatric hospital (9). The present study found that CAP significantly increased the length of hospital stay for inpatients with mental disorders. Comorbid CAP also increased the probability of the patient being admitted to a closed ward or the ICU, as well as the probability of the patient requiring mechanical ventilation during treatment. However, comorbid CAP didn't increase the mortality rate of patients with mental disorders in this general hospital, which was different from the previous reports of psychiatric hospitals (6).

The incidence of CAP among the patients with mental disorders in this study was higher in those aged ≥ 65 years-old, males, and those with a BMI ≤ 18.5 kg/m^2^, which agrees well with previous findings in the general population (8). Therefore, the main analyses were performed following PSM for gender, age, and BMI. After PSM, it was found that the risk factors for CAP in patients with mental disorders admitted to this general hospital were the use of anti-psychotic drugs, especially clozapine, schizophrenia spectrum disorder, organic mental disorder, alcohol consumption, CVD, CCI index ≥ 3, unmarried status and low education level.

The observation that anti-psychotic drug use was associated with increased odds of CAP in inpatients with mental disorders is consistent with previous studies (12). Typical anti-psychotics may enhance the risk of aspiration pneumonia by causing extra-pyramidal side effects such as dyskinesia. Although atypical anti-psychotics are associated with a lower risk of extra-pyramidal side effects, they can cause dry mouth and dysphagia (due to anticholinergic effects) as well as sedation (caused by central nervous system H1 receptor blockade), which are also risk factors for pneumonia. This study found that clozapine, an atypical anti-psychotic drug, was associated with an elevated risk of CAP in inpatients with mental disorders (OR = 3.212). Several previous large-scale investigations have reported that treatment with clozapine is associated with an increased risk of pneumonia (13). Clozapine may be more likely to induce sedation and salivation (14). Clozapine is the antipsychotic drug of choice for the treatment of refractory schizophrenia (15). However, clozapine-treated patients should be monitored carefully.

This study found that inpatients with organic mental disorders or schizophrenia spectrum disorder had a higher incidence of CAP than those with other types of mental disorder. Schizophrenia is a common, chronic, and disabling mental illness (16). A 9-year follow-up study in Taiwan determined that the incidence of pneumonia in patients with schizophrenia was 10.26% and that the incidence density was 11.4/1,000 person-years (17). Many patients with schizophrenia exhibit impairments in social functioning, self-care, and self-control, which may increase the risk of pneumonia. In addition, dysphagia is common in patients with schizophrenia, and this can lead to aspiration pneumonia. Organic mental disorders may have an inherent “organic basis” and hence be associated with physical comorbidities (1), and many patients with organic mental disorders are elderly and have comorbid dementia and cerebrovascular disease, which may increase the risk of pneumonia. Dementia, in particular, is thought to elevate the risk of pneumonia (18).

Previous research showed that polymorbidity (two or more physical comorbidities) was more common in people with mental disorders than in those without mental illness (19). Compared with the general population, patients with mental disorders have a 6–8-fold increase in medical comorbidity, a shorter life expectancy, and a higher mortality (20). The present study found that a CCI index ≥ 3 was a risk factor for CAP in inpatients with mental disorders. The age- and sex-standardized incidence of CVD was found to be higher in patients with mental disorders than in those without mental illnesses (13.5/1,000 person-years vs. 6.3/1,000 person-years), and the highest CVD incidence rates were in patients with bipolar disorder and schizophrenia (21). CVD is a known risk factor for CAP (22). Thus, it is important that clinicians are aware that inpatients with mental disorders and multiple comorbidities are at particular risk of CAP.

This study found that unmarried status was closely related to CAP in inpatients with mental disorders. A population-based case-control study found that the odds of pneumonia-related hospitalization were 33% higher for unmarried people than for married people, and alcoholism-related disorders and other comorbidities appeared to explain the increased risk of pneumonia-related hospitalization in unmarried people (23). Junior high school education was also associated with CAP in inpatients with mental disorders. Prior research has yielded evidence that a higher level of education is associated with a lower risk of major psychiatric disorders and most physical disorders, independent of intelligence (24). In China, low education and being unmarried are both associated with an increased risk of serious mental illness (25). This study found that 6.03% of the inpatients with mental disorders had alcohol misuse disorder, and the consumption of alcohol was identified as a risk factor for CAP in patients with mental disorders. The sedative effects of prolonged alcohol consumption can impair cough and vomiting responses and thereby increase the risk of aspiration.

## Conclusions

The incidence rate of CAP was 1.78% in inpatients with mental disorders. Clozapine, schizophrenia spectrum disorder, alcohol consumption, CVD, CCI index ≥ 3, organic mental disorder, use of antipsychotic drugs, unmarried status, and lower level of education were identified as independent risk factors for CAP in hospitalized patients with mental disorders. These findings provide baseline data for establishing effective prevention strategies of CAP in people with mental disorders.

## Limitations

First, this was a cross-sectional study, and subsequent readmissions and deaths after discharge were not tracked. Second, this study involved only one general hospital, so the findings will need to be further evaluated in a stratified analysis of data from multiple centers of different sizes. Third, the severity of mental disorders on admission could not be assessed in all patients due to the retrospective study design.

## Data availability statement

The original contributions presented in the study are included in the article/supplementary material, further inquiries can be directed to the corresponding authors.

## Author Contributions

JH and GW conceived and designed the experiment. JH performed the research, analyzed the results, wrote the manuscript, analyzed the data, and carried out a literature search. JH, QW, LX, and GW contributed to revising the manuscript. JH and MS collected data. JH and ZL submitted the article. All authors read and approved the final manuscript.

## Funding

This study was supported by the National Natural Science Foundation of China (No. 81571325 and No. 81401117); the Medical Science Advancement Program of Wuhan University (No. TFLC2018001); Design of this study was supported by the National Natural Science Foundation of China (No. 81571325 and No. 81401117).

## Conflict of interest

The authors declare that the research was conducted in the absence of any commercial or financial relationships that could be construed as a potential conflict of interest.

## Publisher's note

All claims expressed in this article are solely those of the authors and do not necessarily represent those of their affiliated organizations, or those of the publisher, the editors and the reviewers. Any product that may be evaluated in this article, or claim that may be made by its manufacturer, is not guaranteed or endorsed by the publisher.
