# Supplementary material for: Risk factors for community-acquired pneumonia among inpatients with mental disorders in a tertiary general hospital
Source: Front Psychiatry. 2022 Jul 22;13:941198. doi: 10.3389/fpsyt.2022.941198 (PMC9354262; doi:10.3389/fpsyt.2022.941198)
Supplement: Supplementary file 1 [file Table_1.pdf]

Supplementary Table 1. Baseline characteristics of the study participants before propensity score matching.

Characteristic	CAP group (n = 301)	Non-CAP group (n = 16,633)	P
Age (years), median (IQR)	50.00 (33.00, 62.00)	31.00 (24.00, 48.00)	<0.001
Gender, Male, n (%)	168 (55.81%)	7063 (42.46%)	<0.001
Education, n (%)			<0.001
Undergraduate and above	41(13.62%)	4713(28.34%)	
College	9 (2.99%)	1834 (11.03%)	
High school or secondary school	94 (31.23%)	4452 (26.77%)	
Junior high school	87 (28.90%)	3804 (22.87%)	
Primary school/illiterate	70 (23.26%)	1827 (10.99%)	
Marital status, n (%)			<0.001
Unmarried	84 (27.91%)	7646 (45.97%)	
Married	182 (60.47%)	7939 (47.73%)	
Widowed or divorced	35 (11.63%)	1047 (6.30%)	
Body mass index, ≤18.5 kg/m^2, n (%)	37 (12.29%)	912 (5.48%)	<0.001
Smoking, n (%)	75 (24.92%)	2294 (13.79%)	<0.001
Alcohol consumption, n (%)	53 (17.61%)	969 (5.83%)	<0.001
Type of mental disorders, n (%)			<0.001

Organic mental disorder	61 (20.27%)	662 (3.98%)	
Schizophrenia spectrum disorder	100 (33.22%)	3466 (20.84%)	
Mood affective disorder	97 (32.23%)	9186 (55.23%)	
Other	43 (14.29%)	3319 (19.95%)	
Age at mental disorder onset (years), median (IQR)	36.00 (24.00, 57.00)	27.00 (20.00, 42.00)	<0.001
Duration of mental disorder (years), median (IQR)	4.00 (0.20, 10.00)	2.00 (0.30, 6.00)	<0.001
Family history of mental disorder, *n* (%)	36 (11.96%)	2491 (14.98%)	0.146
Duration of treatment (years), median (IQR)	1.00 (0.00, 7.00)	0.50 (0.00, 3.00)	<0.001
Duration of antipsychotic drug use (years), median (IQR)	0.20 (0.00, 5.00)	0.00 (0.00, 1.00)	<0.001
Poor adherence to recent therapy[a], *n* (%)	37 (12.29%)	2174 (13.07%)	0.690
Number of antipsychotic drugs currently used, *n* (%)			<0.001
0	161 (53.49%)	12299 (73.94%)	
1	79 (26.25%)	2908 (17.48%)	
2/3/4	61 (20.27%)	1426 (8.57%)	
Current use of clozapine, *n* (%)	64 (21.26%)	669 (4.02%)	<0.001
Cholinesterase inhibitor, use *n* (%)	23 (7.64%)	730 (4.39%)	0.007
Charlson comorbidity index, *n* (%)			<0.001
0–1 points	145 (48.17%)	14433 (86.77%)	

2 points	38 (12.62%)	1159 (6.97%)	
3 points and above	118 (39.20%)	1041 (6.26%)	
Cerebrovascular disease, *n* (%)	88 (29.24%)	907 (5.45%)	<0.001
Cardiovascular disease, *n* (%)	41 (13.62%)	174 (1.05%)	<0.001
Diabetes mellitus, n (%)	39 (12.96%)	457 (2.75%)	<0.001

Note: [a] Failure to take the medication regularly or medication discontinued without authorization during the past 1 month. CAP: community-acquired pneumonia; IQR: interquartile range.
